# PTSD IS ASSOCIATED WITH LONGITUDINAL CHANGES IN DOMAIN-SPECIFIC COGNITIVE FUNCTION IN OLDER ADULTS

**DOI:** 10.1093/geroni/igad104.3542

**Published:** 2023-12-21

**Authors:** Karen Lawrence, Hannah Speaks, Erin Abner, Frederick Schmitt, Jennifer Vasterling, Brian Smith, Suzanne Segerstrom

**Affiliations:** University of Kentucky, Lexington, Kentucky, United States; University of Kentucky, Lexington, Kentucky, United States; University of Kentucky, Lexington, Kentucky, United States; University of Kentucky, Lexington, Kentucky, United States; Boston University Chobanian & Avedisian School of Medicine, Boston, Massachusetts, United States; Boston University Chobanian & Avedisian School of Medicine, Boston, Massachusetts, United States; Oregon State University, Corvallis, Oregon, United States

## Abstract

Posttraumatic stress disorder (PTSD) is associated with an increased risk for all-type dementia yet whether PTSD is associated with domain-specific cognitive decline, that may lead to dementia, is unknown. Using existing data from the National Alzheimer’s Coordinating Center, we tested the hypothesis that PTSD is associated with cognitive decline in episodic memory, working memory and attention, and executive function. After exclusion for history of dementia, stroke, Down syndrome, Parkinson’s and Alzheimer’s diseases, schizophrenia, and neurodevelopmental disorders, 130 (1.25%) participants with PTSD and 10,261 (98.75%) without PTSD remained. Prior to analysis, inverse probability of treatment weighting was used to adjust for confounding. Inverse probability estimates were based on PTSD status and conditional on covariates for age, sex, race, ethnicity, APOE ɛ4 status, anxiety, depression, TBI status, diabetes, and hypertension. Generalized estimating equations (GEE)-type linear regression models with robust standard errors were fit to the weighted data for z-scores for each cognitive domain. During the study, unexpectedly, PTSD was associated with an additional .043 standard deviations of improvement in episodic memory (b = .043, 95%CI: .020, .066) compared to the same population without PTSD. Expectedly, PTSD was associated with an additional .143 standard deviations of decline in working memory and attention (b = -.143, 95% CI: -.225, -.062) and an additional .415 standard deviations of decline in executive function (b = -.415, 95%CI: -.563, -.267). Investigations are ongoing to probe the unexpected finding for episodic memory. These findings suggest that PTSD should potentially be a target for early prevention of cognitive decline.

